# Spatial frequency preferences of representations of indoor and natural scene categories in scene-selective regions under different conditions of contrast

**DOI:** 10.3389/fnins.2025.1534588

**Published:** 2025-02-07

**Authors:** Yuanyuan Zhang, Qiaomu Miao, Baolin Liu

**Affiliations:** ^1^School of Computer and Communication Engineering, University of Science and Technology Beijing, Beijing, China; ^2^College of Intelligence and Computing, Tianjin University, Tianjin, China

**Keywords:** spatial frequency, multivoxel pattern analysis, luminance contrast, scene-selective region, natural scene, indoor scene

## Abstract

**Introduction:**

Scene-selective regions were shown to be significantly affected by spatial frequencies (SF) and have different sensitivities to low spatial frequencies (LSF) and high spatial frequencies (HSF). However, previous studies mainly focused on the neural activations or the neural patterns in a single SF band.

**Methods:**

To investigate the extent to which the information of a single SF is used in scene category representations, we not only decoded the scene categories in each SF, but also used the neural patterns to LSF or HSF to decode the patterns to non-filtered (NF) scenes based on fMRI data using multivoxel pattern analysis (MVPA). As luminance contrast was shown to follow statistical regularities along with SF, we performed the decoding analyses separately in two conditions of contrast where the contrast of LSF and HSF was unmodified or equalized.

**Results:**

The results showed distinct SF preferences in the two contrast conditions, showing that luminance contrast has a significant role in SF processing. In addition, we also performed the above analyses only within natural and indoor scenes, respectively. The results showed the scene-selective regions were more efficient in distinguishing natural scene categories in LSF, and the LSF was preferentially used along with high luminance contrast in recognition of natural scenes. On the other hand, humans preferentially used HSF information in distinguishing indoor scenes.

**Discussion:**

This distinct SF preferences maybe caused by the different aspects of information conveyed by LSF and HSF, as well as the different strategies of spatial perception in natural and indoor scenes recognition.

## Introduction

Complex visual scenes can be interpreted in different levels of properties, including objects, spatial layouts, as well as low-level properties, such as color, orientation and spatial frequencies (SF). Previous research mainly focused on the primary visual cortex (V1) in the processing of low-level visual scene properties, which showed sensitivities to these properties such as spatial frequency, orientations, and luminance contrast (Furmanski and Engel, 2000; Boynton, 2005; Tanaka and Sawada, 2022; Schuurmans et al., 2023). The processing of high-level categorical information of scenes was localized and mainly researched on several occipitotemporal brain regions, including the parahippocampal place area (PPA), retrosplenial cortex (RSC) and occipital place area (OPA) (Epstein and Kanwisher, 1998; Bar and Aminoff, 2003; Yichen and Sheng, 2024), which were typically called “scene-selective regions.” The scene-selective regions showed higher activations in scene viewing compared to viewing other categories of visual stimuli (Epstein and Kanwisher, 1998; O'Craven and Kanwisher, 2014). Multivoxel pattern analysis (MVPA) also showed distinctive patterns of neural responses in these regions to different categories of scenes in the form of photographs and line drawings (Walther et al., 2009; Walther et al., 2011; Brandman and peelen, 2019). In addition, scene-selective regions have also been indicated in the processing of spatial layouts of scenes, such as spatial geometry and expanse (Russell et al., 2003; Kravitz et al., 2011; Mccormick et al., 2021). The PPA has also been indicated in the processing of object-related information, including space diagnosticity, object co-occurrence, and contextual associations (Cant and Xu, 2012; Nestmann et al., 2022), showing greater activations for scenes with incongruent objects (Goh et al., 2004; Rémy et al., 2014). These studies showed the scene-selective regions involve in the processing of mid-level scene components, not solely in the processing of high-level semantic information.

However, recent findings have shown that the neural responses in scene-selective regions can be significantly affected by low-level properties, such as SF, orientations and rectilinearity (Rajimehr et al., 2011; Nasr et al., 2014; Kauffmann et al., 2015a,b; Kreiman and Serre, 2020). SF was especially highlighted in causing different levels of neural activations in scene-selective cortices in different types of filtering (Peyrin et al., 2004; Rajimehr et al., 2011; Kauffmann et al., 2015a,b; Perfetto et al., 2020). Nevertheless, previous univariate analyses have showed diverse results. Several recent studies suggest higher activations in scene-selective regions to high spatial frequency (HSF) scenes (Rajimehr et al., 2011; Canário et al., 2016; Berman et al., 2017), while there were also studies showing higher activations to low spatial frequency (LSF) scenes (Peyrin et al., 2004; Schettino et al., 2011). Although numerous studies have investigated the neural activations to different SF, only a few studies have focused on the patterns of neural responses in scene representations activated by different SF. A recent study using MVPA found that neural patterns could be better decoded by SF than by image content (Watson et al., 2016), suggesting that neural patterns could also be affected by SF. A latest MVPA decoded different scene categories within each SF band and observed higher accuracies in HSF (Berman et al., 2017). However, the decoding within a single SF could only reflect the abilities to dissociate scene categories in a certain type of SF. High accuracy in HSF does not necessarily imply that the same information is prioritized in natural scene viewing. Therefore, it is of greater importance to investigate the extent to which the category-specific neural patterns to scenes in a certain SF is represented in the patterns to unfiltered scenes by performing cross-decoding analysis between different SF.

Conversely, prior studies predominantly employed luminance contrast equalization in SF processing research. However, luminance contrast across SF scenes exhibits statistical regularities, approximately adhering to a 1/fα function, leading to a significantly higher contrast in LSF images compared to HSF after filtering. As a result, using contrast equalization may result in irrelevant analyses of the SF processing of visual scenes (Kauffmann et al., 2015a,b). In order to reveal the impact of luminance contrast on SF processing, one previous study has researched on the neural activations with and without equalization of root mean square (RMS) contrast in a scene categorization task (Kauffmann et al., 2015a,b). The RMS contrast, which corresponds to the standard deviation of luminance values, has been proven to be the most reliable measure of the visibility of broadband filtered images (Liu-Shuang et al., 2022). Results showed distinct SF preferences in PPA and RSC, which confirmed the impact of luminance contrast on the sensitivities to different SF in scene-selective regions. Consequently, it is vital to investigate the effect of luminance contrast on SF processing in representation of scene categories. In addition, different SF mainly convey different aspects of information in visual scenes. As LSF information mainly captured the coarse blobs and patches, and HSF information mainly revealed the detailed edges, the global information of scenes is principally conveyed by LSF, while local and detailed information is mostly conveyed by HSF. Studies have shown that LSF activated the brain regions related to peripheral vision, while HSF activated the regions dedicated to foveal vision (Sasaki et al., 2001; Henriksson et al., 2007; Canário et al., 2016), but there was also an interaction between low and high SF information processing (Revina et al., 2017; Takeda et al., 2021; Schuurmans et al., 2023). In scene recognition, humans typically are more involved in local space processing for indoor scenes than natural scenes, which may rely more on global perception (Epstein, 2005; Henderson et al., 2007). Computer vision studies have also showed that indoor scenes have a greater proportion of horizontal and vertical edges than natural scenes, and the differentiation between man-made scene categories reside mainly in the relationship between these edges (Torralba and Oliva, 2003). Natural scenes, such as beach and mountain, typically contain more LSF information. The distinct preferences for spatial SF in neural patterns may arise from the differences in spatial frequency distribution between natural and indoor scenes, as well as variations in perceptual mechanisms.

In this study, we mainly focused on the SF preferences in the neural representations of visual scene categories in scene-selective regions. Participants observed scenes under various SF conditions, encompassing non-filtered (NF) scenes, LSF scenes, and HSF scenes in a block-designed experiment. In order to investigate the impact of luminance contrast on SF processing, we referred to the design in (Kauffmann et al., 2015a,b), and set 2 conditions of luminance contrast, LUM and RMS. In LUM condition, the contrast of LSF and HSF scenes were unmodified after filtering, while in RMS condition, all images were normalized to the same luminance contrast. In these two conditions, we used MVPA to decode the scene categories within each SF to investigate the sensitivities of the scene-selective regions to the scene category information in each SF, and further decoded the neural patterns to NF scenes by that of LSF and HSF scenes to investigate the SF preferences in actual scene viewing. The classification accuracies were statistical analyzed in each contrast condition to see if the SF preferences differ between the two contrast conditions. In addition, in order to know whether the role of SF in category representations differ between indoor and natural scenes, we performed the above decoding analyses within indoor and natural scene categories, respectively, and analyzed the SF preferences.

## Materials and methods

### Participants

Twenty-seven right-handed healthy subjects (age: 18–32, 14 females) with normal or corrected-to-normal vision participated in this study. This study was carried out in accordance with the recommendations of Institutional Review Board (IRB) of Tianjin University. The protocol was approved by the IRB of Tianjin University. All subjects gave written informed consent in accordance with the Declaration of Helsinki. All subjects were compensated for their time after the experiment.

### Experimental stimuli

**Figure 1:**
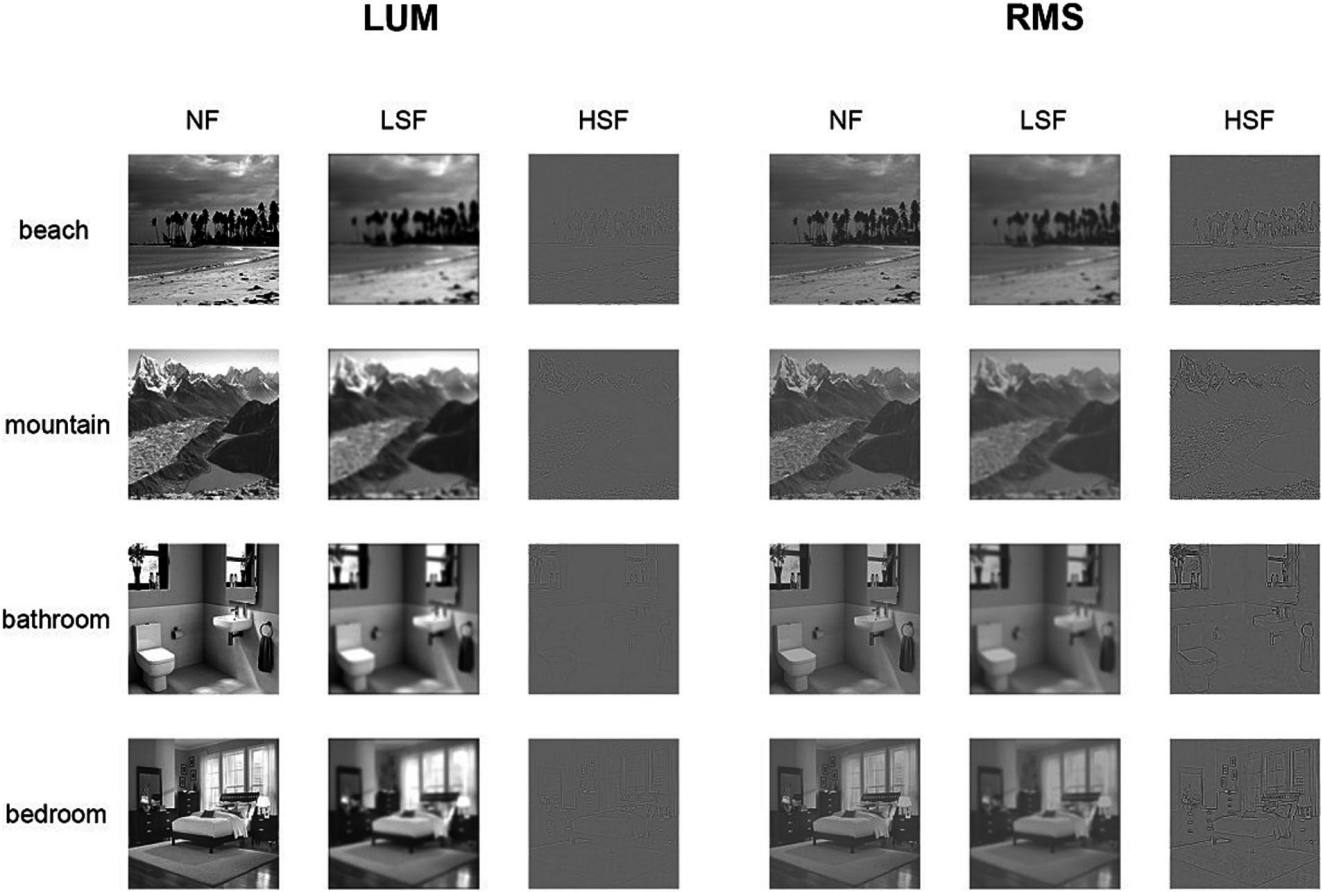
Experimental stimuli. The whole set of stimuli consists of 4 scene categories: beach, mountain, bathroom and bedroom, the first two are natural scenes, and the latter two are indoor scenes. Each category comprises 120 original images, which are used as the non-filtered scenes (NF) in the LUM condition. The LSF and HSF images are obtained by removing the SF content above 1 cpd and below 5cpdin SF filtering. In LUM condition, the contrast of LSF and HSF images are left unchanged. In the RMS condition, the luminance contrast of all images was normalized to an RMS contrast of 0.12. Note that for illustration purpose, the image displayed is substantially smaller in size than the actual stimuli in the fMRI experiment.

The visual stimuli were grayscale images from 2 natural scene categories (beach, mountain) and 2 indoor scene categories (bathroom, bedroom). Each scene category comprised 120 images. The original images were downloaded from the Internet and were resampled to a size of 600 × 600 pixels. Before spatial frequency filtering, all images were equalized in mean luminance and contrast to obtain a mean luminance of 0.5 and an RMS contrast of 0.23 (corresponding to a mean gray scale of 128 and an RMS contrast of 60 on a 256 gray-level scale) using the SHINE toolbox (Willenbockel et al., 2010). These images were used as the non-filtered scenes (NF) in the LUM condition. Afterwards, spatial frequency filtering was performed by first performing Fourier transform on the images, then using Gaussian function as the transfer function, adjusting its parameters as needed to achieve low-pass or high pass filtering, then multiplying the transfer function with the frequency domain image, and finally performing inverse Fourier transform to convert the processed frequency domain image back to the spatial domain, obtaining the filtered image. The LSF scenes were generated by removing the spatial frequency content above 1 cycles per degree (cpd), and the HSF scenes by removing the spatial frequency content below 5 cpd at FWHM. The cut-off frequencies were selected consistent with a previous study on the SF processing of scenes and faces (Rajimehr et al., 2011). With 4 scene categories and 3 SF conditions, we got 4 × 3 = 12 conditions of images for each contrast condition. The contrast of the images in the 3 SF conditions were then modified to form the 2 contrast conditions. In the LUM condition, the contrast of the images were left unchanged. In the RMS condition, the images in all 3 SF conditions were normalized to an RMS contrast of 0.12 (30 on a gray-level scale). This contrast value was chosen between the contrast value of LSF and HSF images in the LUM condition, to avoid affecting one SF condition more than the other. The example stimuli in the 2 contrast conditions were displayed in Figure 1. Table 1 displayed the luminance contrast calculated in each category and SF condition in LUM or RMS. The images were presented through the high-resolution stereo 3D glasses of the VisuaStim Digital MRI Compatible fMRI system, subtending approximately 22.5° × 22.5° of visual angle.

**Table 1:** Mean luminance contrast of each scene category in each contrast condition.

SF	LUM				RMS			
	Beach	Mountain	Bathroom	Bedroom	Beach	Mountain	Bathroom	Bedroom
NF	0.23 ± 0.00	0.23 ± 0.00	0.23 ± 0.00	0.23 ± 0.00	0.12 ± 0.00	0.12 ± 0.00	0.12 ± 0.00	0.12 ± 0.00
LSF	0.24 ± 0.02	0.24 ± 0.03	0.23 ± 0.02	0.23 ± 0.01	0.12 ± 0.00	0.12 ± 0.00	0.12 ± 0.00	0.12 ± 0.00
HSF	0.04 ± 0.01	0.05 ± 0.01	0.04 ± 0.01	0.04 ± 0.01	0.12 ± 0.00	0.12 ± 0.00	0.12 ± 0.00	0.12 ± 0.00

Mean luminance contrast (mean ± standard deviation) for each scene category, in 3 SF conditions (NF, LSF, and HSF) for each contrast condition (LUM and RMS). Luminance values were normalized in the range [0 1].

### Experimental design

The main experiment was conducted in two separate sessions, in which the visual scene images in one contrast condition: LUM or RMS were presented. Each session consisted of 4 runs, and each run was composed of 12 blocks, lasting for 8 min and 10s. Each block lasted for 30s, in which 30 images from the same scene category and the same SF condition were presented. Each image was presented for 500 ms, interleaved with a 500 ms gray screen with a fixation cross in the center, which was equal in mean luminance to the scene images. The order of the stimulus blocks in each run and image presentations in each block was randomized. Blocks were separated by a 10-s baseline of fixation, in which the same gray screen used in the interstimulus interval was presented. Subjects performed a “one-back” repetition detection task to maintain attention in which they were asked to press a button when two consecutive images were identical.

An additional localizer run was performed after the main experiment in the first session. The design of the localizer run refers to the procedures in (Macevoy and Epstein, 2011). Subjects viewed four kinds of color images: scenes, faces, objects, and phase-scrambled objects in a total of 16 blocks. Images had a resolution of 600 × 600 pixels (corresponding to approximately 12° × 12° of visual angle). Each block presented 25 different images of a single kind consecutively, so each kind of stimuli occupied for 4 blocks. Each image was presented for 800 ms. A central fixation cross was superimposed on all images. Blocks were separated by a 10-s interval of baseline. The localizer run lasted for 8 min 10s.

### Behavioral experiment

To confirm that the spatial frequency filtering process did not disrupt the participants’ abilities to perceive the categories of the scenes, we conducted an additional behavioral experiment. Another 22 participants (12 females, age 22–32) performed a scene categorization task on the visual stimuli which were used in the fMRI experiment. The behavioral experiment consist of two separate runs, in which the images in the LUM or RMS condition were presented. In each run, there are 12 conditions (3SF × 4 scene categories) as the fMRI experiment, and in each condition, 10 images were randomly selected from the whole stimuli set in the fMRI experiment. The original images of all the selected images were not repeated. Each run consisted of 120 trials. In each trial a fixation cross was presented for 1,000 ms, and an image was presented for 500 ms, as in the fMRI experiment, and followed by a gray screen for 2,500 ms or until the participant made a response. The participants were instructed to press a button to indicate the scene category of the presented image as quickly and as accurately as possible. A chin rest was used to maintain the viewing distance, making the visual angle subtend approximately 22.5°, as in the fMRI experiment. Participants’ responses were recorded using the E-prime software.

### fMRI data preprocessing

Data were preprocessed using SPM8.^1^ Five volumes at the beginning of each run were discarded before the following data processing. Functional images were corrected in slice timing and motion corrected with respect to the first volume of each run with a six-parameter rigid body transformation. Structural T1-weighted images were co-registered to the mean functional image for each participant and then segmented into white matter, gray matter and cerebral spinal fluid (CSF). The generated mapping parameters were then used to spatially normalize the realigned images into the standard Montreal Neurological Institute (MNI) space at 3 × 3 × 3 mm^3^ (Liang et al., 2017; Liang et al., 2018; Yang et al., 2018). Only the data in the localizer run were spatially smoothed using a 6 mm full width half maximum (FWHM) Gaussian kernel.

### MRI acquisition

All functional data were acquired using a 3.0 T Siemens scanner equipped with an 8-channel head coil at Yantai Affiliated Hospital of Binzhou Medical Univeristy. T2*-weighted images were acquired using an echo-planar image (EPI) sequence (TR = 2,000 ms, TE = 30 ms, voxel size = 3.1 × 3.1 × 4.0 mm^3^, matrix size = 64 × 64, slices = 33, slice thickness = 4 mm, slice gap = 0.6 mm, flip angle (FA) = 90°). T1-weighted anatomical images were acquired with a three-dimensional magnetization-prepared rapid-acquisition gradient echo (3D MPRAGE) sequence (TR = 1900 ms, TE = 2.52 ms, TI = 1,100 ms, voxel size = 1 × 1× 1 mm^3^, matrix size = 256 × 256, FA = 90°). Foam pads and earplugs were used to reduce the head motion and scanner noise.

### ROI definition

**Figure 2:**
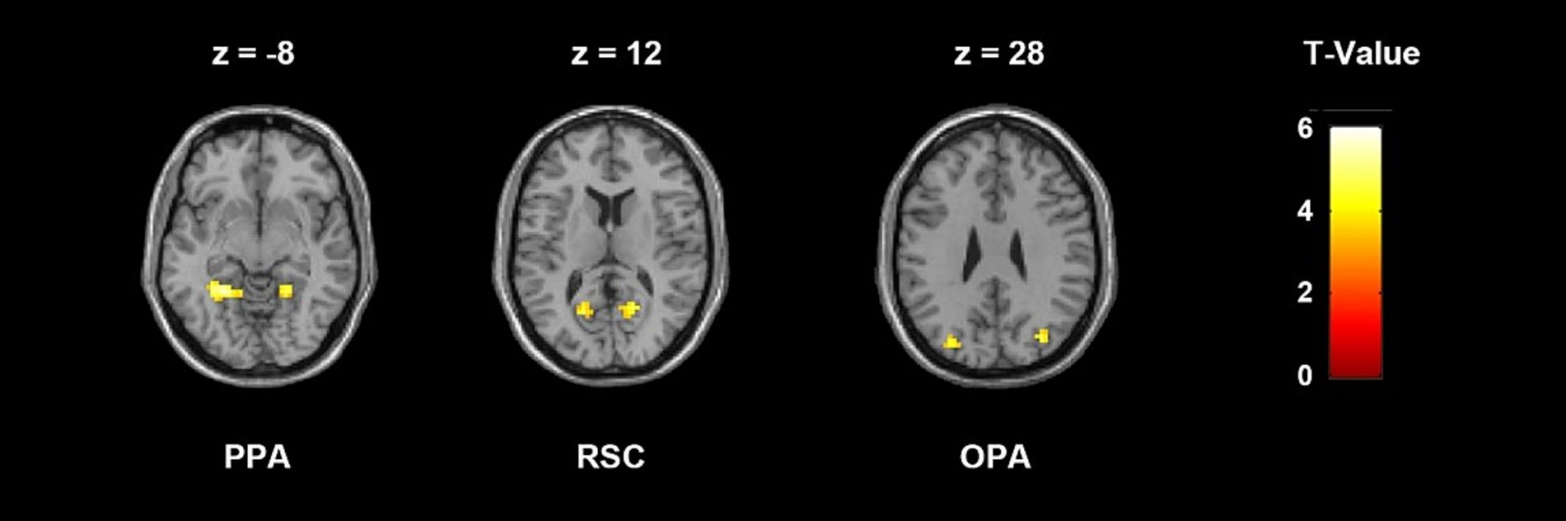
ROI localization results of a representative subject, thresholded at uncorrected *p* < 0.005 with a cluster extent *k* = 20 voxels. Peak voxel was selected in each cluster and each ROI was created by drawing an 8 mm sphere around that voxel. The MNI coordinates of the peak voxels of this subject are: LPPA, −24 −48 −9; RPPA, 21 −48 −12; LRSC, −18 −63 12; RRSC, 18 −60 12; LOPA: −30 −78 27; ROPA: 33 −75 27.

Scene-selective functional regions PPA, RSC and OPA were defined bilaterally from the localizer run, all of which were defined using the contrast of scenes>faces + objects, and drawing an 8 mm radius sphere around the peak voxel in each hemisphere. Most clusters were thresholded at uncorrected *p* < 0.005 with a cluster extent of *k* = 20 voxels. The thresholds were relaxed in few regions in certain subjects up to uncorrected *p* < 0.05. The peak voxel of PPA was selected in the posterior parahippocampal-collateral sulcus region, RSC in the restrosplenial cortex-posterior cingulate-medial parietal region, and OPA in the transverse occipital cortex. The RSC region was not identified in two subjects. Figure 2 shows the localization results of a representative subject.

### Univariate analysis

We first performed a univariate analysis on the ROIs by calculating the percent signal change in the scene-selective ROIs in all SF conditions using the marsbar software (http://marsbar.sourceforge.net/) in LUM and RMS conditions to investigate differences in neural activations between different SF conditions when the luminance contrast was unmodified or equalized. A 2 (contrast condition: LUM vs. RMS) × 2 (SF: LSF, HSF) repeated-measure analysis of variance (ANOVA) was performed on the percent signal change to examine the main effect of SF and the interactions between SF and contrast. Due to that the contrast of LSF images was decreased and that of HSF images was increased in the RMS condition, we did not manifest the main effect of contrast in this study.

### Multivoxel pattern analysis

Two types of MVPA analyses were performed to examine the SF selectivity in scene-selective regions, Single-SF decoding analysis and Cross-SF decoding analysis.

### Decoding in single SF

Firstly, we performed a decoding analysis in each single SF condition to examine the abilities to distinguish scene categories in each SF for each scene-selective cortex. For each contrast session, we extracted the unsmoothed functional images in each SF condition. Intensity images were z-score normalized for each functional run in order to eliminate the baseline shifts in different runs. A support vector machine (SVM) classifier was implemented using lib-SVM to classify the scene categories in each SF condition for each scene-selective ROI. The time courses were shifted 2 TRs to account for the hemodynamic lag. Decoding was performed separately for each SF condition (NF, LSF, HSF). Each volume in each block was used as one sample and labeled with its corresponding scene category, so there’re 15 × 4 = 60 samples for each category in each SF condition. A leave-one-run-out cross-validation was adopted, using each of the 4 runs as test data, and the other 3 runs as training data. The accuracies in each iteration were averaged to represent the decoding accuracy in each SF for each subject. A one-tailed one-sample *t*-test was performed to test the significance against chance (0.25) on the decoding accuracies. Two-tailed paired *t*-test between the accuracies of the LSF and HSF conditions were also performed to examine the differences in the abilities to distinguish scene categories between the 2 SF. The above decoding procedure was conducted identically and separately for the two contrast conditions. A 2 (contrast condition: LUM vs. RMS) × 2 (SF: LSF, HSF) repeated-measure analysis of variance (ANOVA) was performed on the classification accuracies to examine the main effect of SF and the interactions between SF and contrast. In addition, the same decoding analysis was applied specifically to only the natural or indoor scenes to examine whether the decoding results differ between the broad scene categories.

### Decoding across SF

A Cross-SF decoding was also performed to specially investigate the degree of representations of LSF or HSF information in NF scenes in order to inspect the spatial frequency preferences of scene representations in scene-selective cortex. The decoding process was similar to the single-SF decoding experiment, except that the SVM classifiers were trained on either the LSF or HSF data, and tested on the NF data, to examine the extent to which the information in either SF was represented when the original scene images were viewed. A one-tailed one-sample *t*-test was performed to test the significance against chance (0.25) on the decoding accuracies. The spatial frequency preferences in normal scene viewing were determined by the two-tailed paired *t*-tests between the classification accuracies when the classifier was trained with functional images in LSF or HSF condition. As in the experiment of decoding in single SF, the cross decoding procedure was conducted identically and separately for the two contrast conditions. A 2 × 2 repeated-measure ANOVA was also performed. The above procedure was additionally conducted specifically to the natural or indoor scene categories.

## Results

### Univariate analysis results

**Figure 3:**
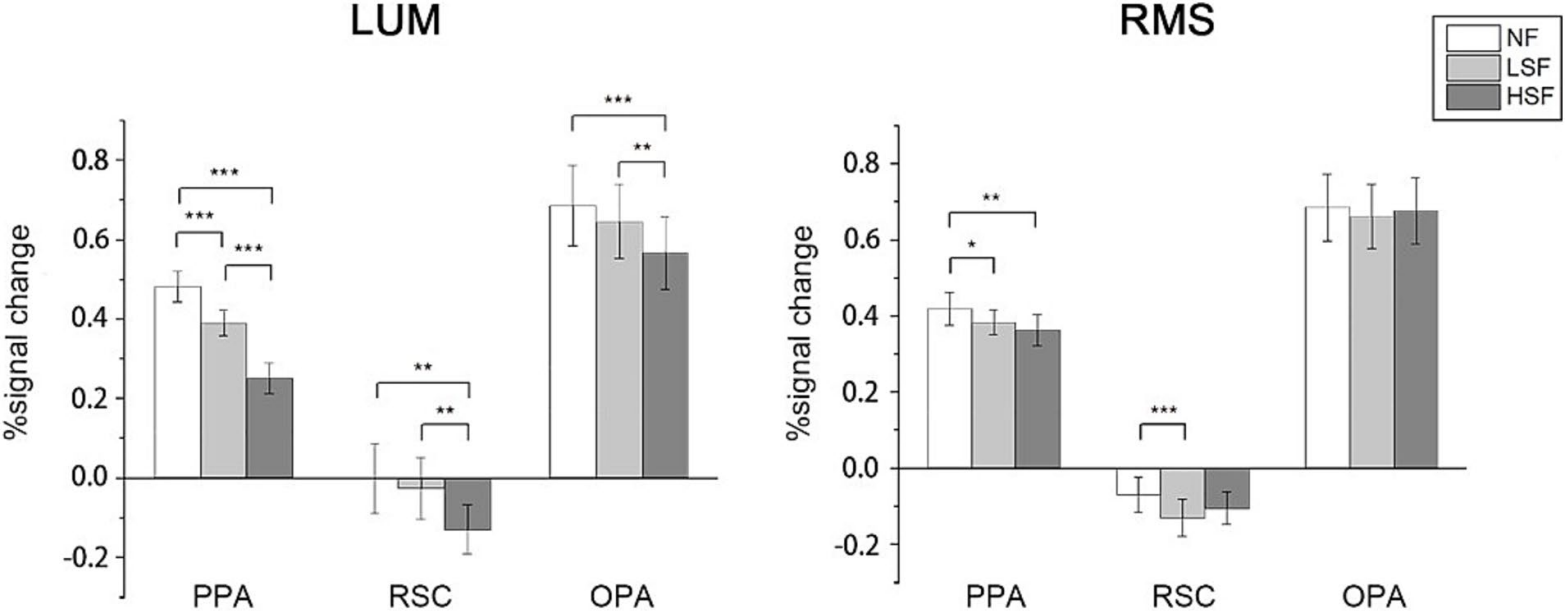
Univariate analysis results. For each condition of contrast (LUM or RMS), percent signal change was calculated in each scene-selective ROI in NF, LSF, and HSF conditions. Error bars indicate standard errors; *p < 0.05, **p < 0.01, ***p < 0.001.

In univariate analysis, percent signal change was calculated in the 3 scene-selective regions in NF, LSF and HSF conditions regardless of scene categories. In LUM, two-tailed paired *t*-test on all ROIs showed significantly higher activations in LSF than HSF scenes were observed (PPA: *t*(26) = 5.854, *p* < 0.001; RSC: *t*(24) = 3.742, *p* = 0.001; OPA: *t*(26) = 3.667, *p* = 0.001), and high activations in NF than HSF (PPA: *t*(26) = 9.344, *p* < 0.001; RSC: *t*(25) = 3.430, *p* = 0.002; OPA: *t*(26) = 4.578, *p* < 0.001). Higher activations were also observed in NF than LSF in PPA and OPA (PPA: *t*(26) = 8.683, *p* < 0.001; OPA: *t*(26) = 2.601, *p* = 0.015). In RMS conditions, two-tailed paired *t*-test showed that no significant differences in activations between LSF and HSF was observed, although results showed higher activations in NF than LSF in PPA (*t*(26) = 2.141, *p* = 0.042) and RSC (*t*(24) = 4.426, *p* < 0.001), and higher activations in NF than HSF in PPA (*t*(26) = 3.366, *p* = 0.002). Two-way ANOVA on the signal change showed significant main effect in SF in PPA (*F*(1, 26) = 16.571, *p* < 0.001) and RSC (*F*(1, 24) = 4.851, *p* = 0.037), and significant interactions in all 3 ROIs (PPA: *F*(1, 26) = 29.397, *p* < 0.001; RSC: *F*(1, 24) = 20.693, *p* < 0.001; *F*(1, 26) = 16.555). RSC showed deactivations in all conditions, possibly because of its location in the default mode network (DMN), which shows deactivations in attention-demanding tasks (Raichle et al., 2001; Singh and Fawcett, 2008). The mean percent signal change is showed in Figure 3. Two-way ANOVA showed significant main effect in SF in PPA and RSC (PPA: *F*(1, 26) = 16.571, *p* < 0.001; RSC: *F*(1, 24) = 4.851, *p* = 0.037), and significant interactions between SF and contrast in all 3 ROIs (PPA: *F*(1, 26) = 29.397, *p* < 0.001; RSC: *F*(1, 24) = 20.693, *p* < 0.001; *F*(1, 26) = 16.555).

### Single-SF decoding results

One-tailed one-sample *t*-test showed that when the decoding was applied to all 4 categories, the scene categories could be significantly decoded in all SF in the LUM condition for all the 3 ROIs: PPA (NF: *t*(26) = 5.590, *p* < 0.001; LSF: *t*(26) = 5.735, *p* < 0.001; HSF: *t*(26) = 3.282, *p* = 0.015), RSC (NF: *t*(24) = 4.077, *p* < 0.001; LSF: *t*(24) = 4.518, *p* < 0.001; HSF: *t*(24) = 3.956, *p* < 0.001), and OPA (NF: *t*(26) = 4.526, *p* < 0.001; LSF: *t*(26) = 3.852, *p* = 0.001; HSF: *t*(26) = 3.658, *p* < 0.001). Two-tailed paired *t*-tests showed no significant difference in accuracies between different SF. In the RMS condition, the result was similar for all the 3 ROIs (PPA-NF: *t*(26) = 5.008, *p* < 0.001; PPA-LSF: *t*(26) = 8.374, *p* < 0.001; PPA-HSF: *t*(26) = 6.711, *p* < 0.001; RSC-LSF: *t*(24) = 6.444, *p* < 0.001; RSC-HSF: *t*(24) = 2.856, *p* = 0.009; OPA-NF: *t*(26) = 4.357, *p* < 0.001; OPA-LSF: *t*(26) = 4.357, *p* < 0.001; OPA-HSF: *t*(26) = 3.952, *p* < 0.001), except that the decoding was not significant in RSC in the NF condition. Paired *t*-tests showed significantly higher accuracies in LSF than NF (*t*(24) = 4.076, *p* < 0.001). No significant effect was revealed in the 2 way repeated-measure ANOVA on the accuracies from the two contrast conditions.

When the decoding procedure was restricted to natural scenes, in LUM condition, one-tailed one-sample *t*-test showed that the categories could be decoded significantly above chance in NF and LSF conditions in all 3 ROIs (PPA-NF: *t*(26) = 2.369, *p* = 0.013; PPA-LSF: *t*(26) = 4.776, *p* < 0.001; RSC-NF: *t*(24) = 2.385, *p* = 0.013; RSC-LSF: *t*(24) = 3.647, *p* < 0.001; OPA-NF: *t*(26) = 2.114, *p* = 0.022; OPA-LSF: *t*(26) = 3.641, *p* < 0.001). The scenes could not be classified above chance in the HSF condition. Two-tailed paired *t*-test showed significant higher accuracies in NF and LSF conditions than in HSF (NF vs. HSF: *t*(26) = 2.326, *p* = 0.028; LSF vs. HSF: *t*(26) = 3.453, *p* = 0.002) in PPA, and higher accuracy in LSF than HSF in RSC (*t*(24) = 2.116, *p* = 0.045). In the RMS condition, one-tailed one-sample *t*-test showed that the categories could be significantly classified in all SF conditions in PPA (NF: *t*(26) = 2.163, *p* = 0.02; LSF: *t*(26) = 4.921, *p* < 0.001; HSF: *t*(26) = 3.661, *p* < 0.001) and OPA(NF: *t*(26) = 2.111, *p* = 0.023; LSF: *t*(26) = 3.951, *p* < 0.001; HSF: *t*(26) = 2.916, *p* = 0.004), and significantly classified in LSF and HSF conditions in RSC (LSF: *t*(24) = 3.857, *p* < 0.001; HSF: *t*(24) = 2.832, *p* = 0.005). Two-tailed paired *t*-tests revealed significantly higher decoding accuracies in LSF than HSF and NF in PPA (LSF vs. HSF: *t*(26) = 2.314, *p* = 0.029; LSF vs. NF: *t*(26) = 2.679, *p* = 0.013), and significantly higher decoding accuracy in LSF than NF in RSC (*t*(24) = 3.301, *p* = 0.003). Two-way ANOVA revealed significant main effect of SF in all 3 ROIs (PPA: *F*(1, 26) = 14.972, *p* = 0.001; RSC: *F*(1, 24) = 5.564, *p* = 0.027; OPA: *F*(1, 26) = 6.476, *p* = 0.017). Two-tailed paired *t*-tests between LSF and HSF regardless of contrast condition showed significantly higher classification accuracies of LSF than HSF images in all ROIs (PPA: *t*(53) = 4.108, *p* < 0.001; RSC: *t*(49) = 2.378, *p* = 0.021; OPA: *t*(53) = 2.063, *p* = 0.044).

**Figure 4:**
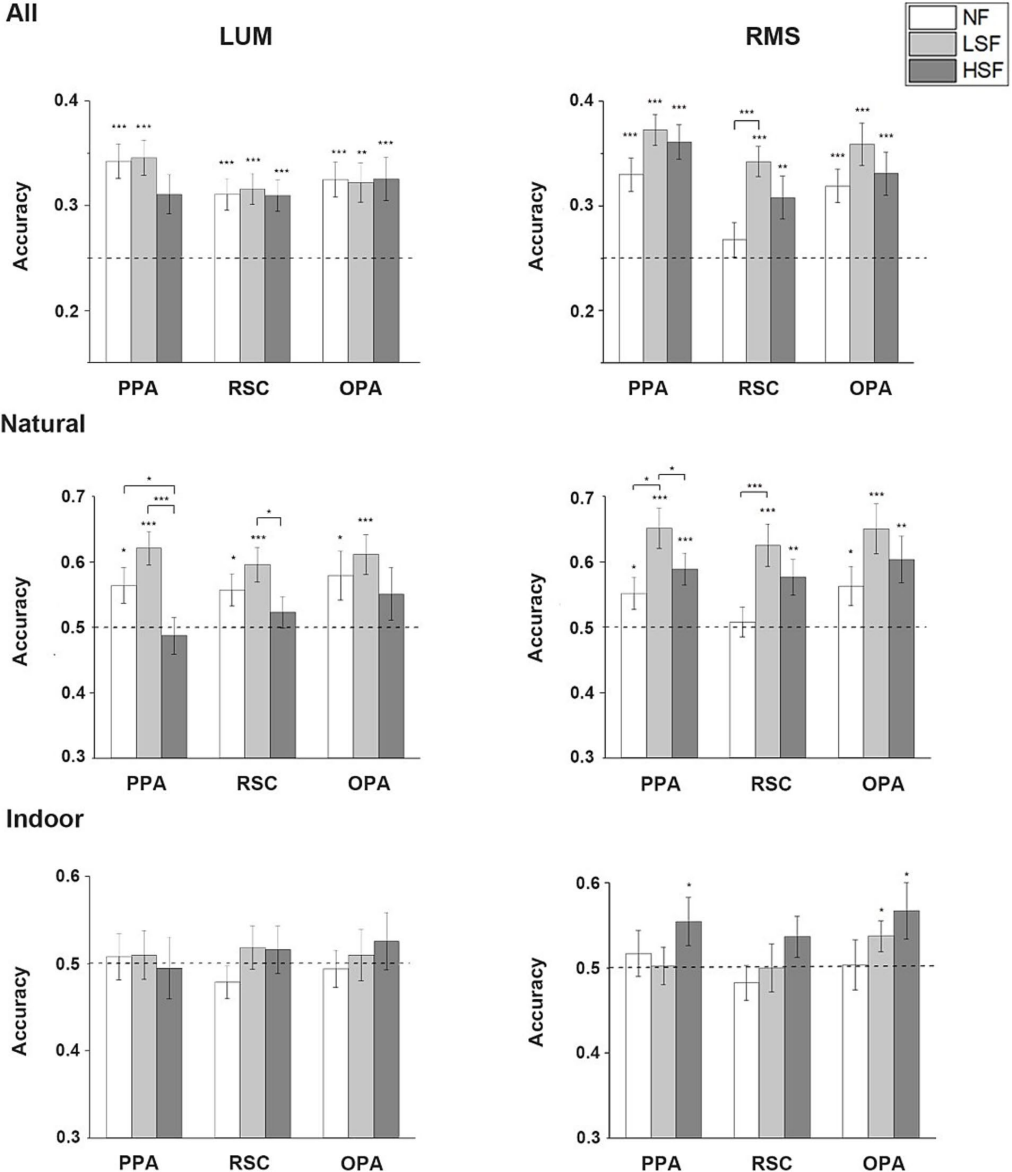
Single-SF decoding results. Mean accuracies across all subjects in the 3 scene-selective ROIs in NF, LSF and HSF are calculated in LUM and RMS conditions when the decoding was performed in all 4 categories, only in natural scenes, and only in indoor scenes. The dashed lines indicate chance level. Error bars indicate standard errors; *p < 0.05, **p < 0.01, ***p < 0.001.

When the decoding was restricted to the indoor scenes, no ROI could significantly classify the indoor scene categories in any SF in LUM condition. However, in RMS condition, one-tailed one-sample *t*-test showed that the indoor scenes could be classified in HSF in PPA (*t*(26) = 1.910, *p* = 0.034) and OPA (*t*(26) = 1.968, *p* = 0.03) significantly above chance, and marginal significantly in RSC (*t*(24) = 1.481, *p* = 0.076). The scenes could also be significantly classified in LSF in OPA (*t*(26) = 1.969, *p* = 0.03). Although the average decoding accuracy in HSF was higher than in LSF, no significant difference was revealed in the paired *t*-tests. No significant effect was revealed in the two-way repeated-measure ANOVA. The average classification accuracies in the Single-SF decoding analysis is showed in Figure 4.

### Cross-SF decoding results

Cross decoding analysis was performed by training the classifier on either the neural responses to LSF and HSF scenes and testing on the responses to NF scenes. When decoding was applied to all 4 scene categories, significant difference was revealed in the classification accuracies between the 2 contrast conditions. In LUM condition, one-tailed one-sample *t*-test showed that the accuracies were significantly above chance (25%) when the classifiers were trained on the neural responses to LSF scenes in PPA and OPA (PPA: *t*(26) = 3.940, *p* < 0.001; OPA: *t*(26) = 1.981, *p* = 0.029), and marginally significant in RSC (*t*(24) = 1.584, *p* = 0.063). Classification accuracies were not significantly above chance in any of the ROIs when the classifiers were trained on the responses to HSF scenes. Two-tailed paired *t*-tests showed significant higher accuracies when the LSF scenes were used than HSF scenes in all 3 ROIs (PPA: *t*(26) = 6.119, *p* < 0.001; RSC: *t*(24) = 3.294, *p* = 0.003; OPA: *t*(26) = 4.880; *p* < 0.001). On the contrary, in the RMS condition, one-tailed one-sample *t*-test showed that the neural responses to NF scenes could all be significantly classified by the responses to HSF scenes (PPA: *t*(26) = 6.782, *p* < 0.001; RSC: *t*(24) = 3.519, *p* = 0.001; OPA: *t*(26) = 5.657, *p* < 0.001). Only PPA could significantly decode the NF scenes by training on the LSF scenes (*t*(26) = 2.429, *p* = 0.011). Two-tailed paired *t*-tests revealed significantly higher classification accuracies when the NF scenes were decoded by HSF scenes than LSF scenes in all 3 ROIs (PPA: *t*(26) = 2.157, *p* = 0.04; RSC: *t*(24) = 2.526, *p* = 0.019; OPA: *t*(26) = 4.537, *p* < 0.001). Significant interaction between SF and contrast was found in the two-way ANOVA on the accuracies from the two contrast conditions (PPA: *F*(1, 26) = 33.988, *p* < 0.001; RSC: *F*(1, 24) = 20.982, *p* < 0.001; OPA: *F*(1, 26) = 45.925, *p* < 0.001).

When the decoding was restricted to natural scene categories, one-tailed one-sample *t*-test showed that the neural responses to NF scenes could be decoded by the responses to LSF scenes in all 3 ROIs in the LUM condition (PPA: *t*(26) = 2.225, *p* = 0.018; RSC: *t*(24) = 2.086, *p* = 0.024; OPA: *t*(26) = 2.106, *p* = 0.023). Two-tailed paired *t*-tests showed that significant higher accuracies were observed compared to training on HSF scenes (PPA: *t*(26) = 6.585, *p* < 0.001; RSC: *t*(24) = 5.607, *p* < 0.001; OPA: *t*(26) = 6.974, *p* < 0.001). Moreover, the accuracy of training on HSF scenes is significantly lower than the chance level. However, the one-tailed one-sample *t*-test results turned to be different in the RMS condition, in which the accuracies were significantly above chance when the classifiers were trained on the responses to HSF scenes in PPA (*t*(26) = 4.471, *p* < 0.001) and OPA (*t*(26) = 2.148, *p* = 0.021). The NF scenes could also be decoded by LSF scenes in PPA, but less significant (*t*(26) = 1.760, *p* = 0.045). No significant difference in accuracies was revealed in the two-tailed paired *t*-tests. All 3 ROIs showed significant main effect in SF (PPA: *F*(1, 26) = 11.141, *p* = 0.003; RSC: *F*(1, 24) = 10.391, *p* = 0.004; OPA: *F*(1, 26) = 15.173, *p* = 0.001) and interaction (PPA: *F*(1, 26) = 32.976, *p* < 0.001; RSC: *F*(1, 24) = 13.661, *p* = 0.001; OPA: *F*(1, 26) = 28.882; *p* < 0.001) in the two-way ANOVA. Two-tailed paired *t*-tests regardless of the contrast manipulation showed significant higher accuracies when the classifiers were trained by LSF than HSF data (PPA: *t*(53) = 2.902, *p* = 0.005; RSC: *t*(49) = 3.056, *p* = 0.004; OPA: *t*(53) = 2.982, *p* = 0.004), but due to the respective results in each contrast condition and the interaction revealed, we think this may mainly be caused by the relative large differences in accuracies in the LUM condition.

**Figure 5:**
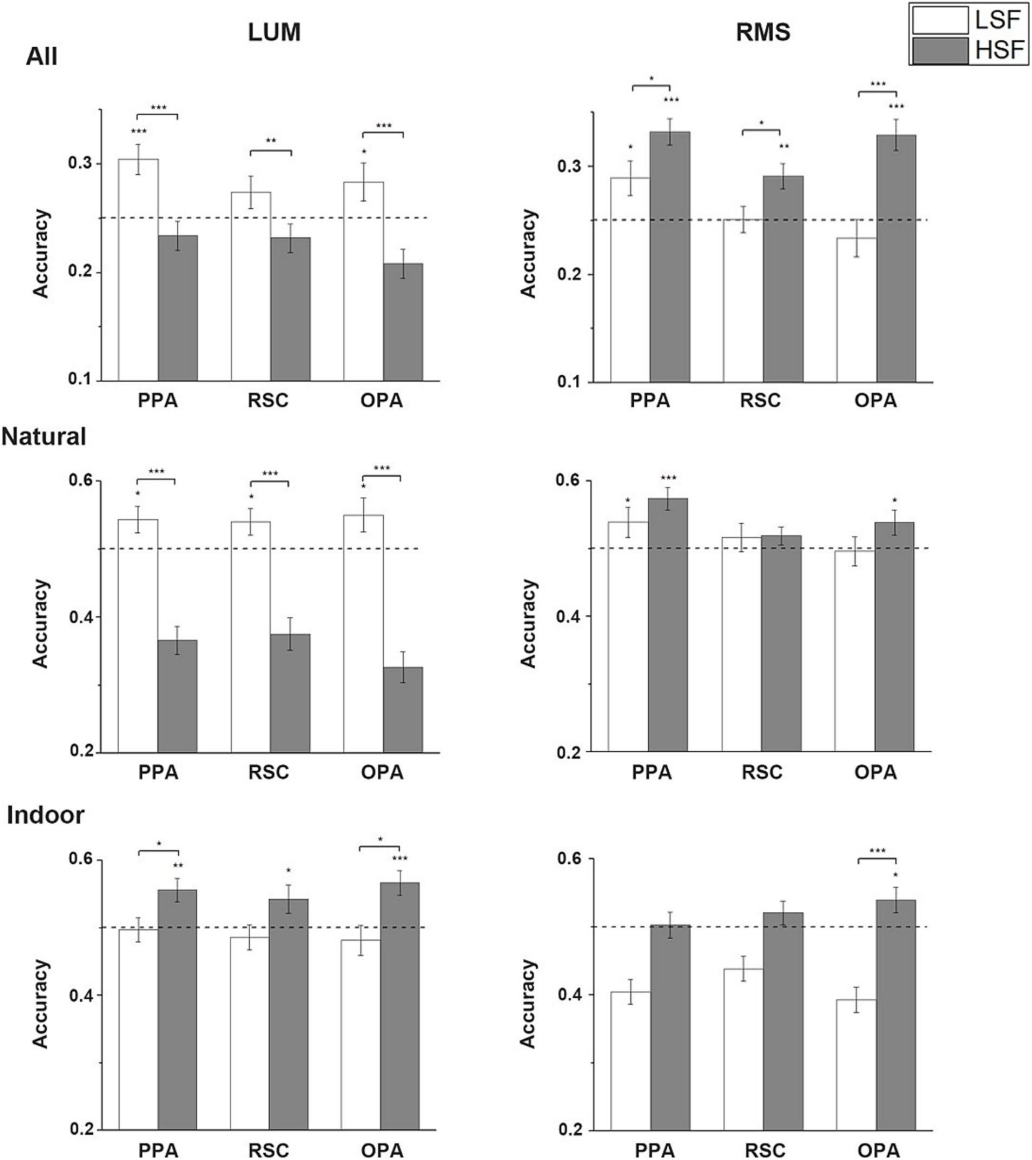
Cross-SF decoding results. Mean accuracies across all subjects in the 3 scene-selective ROIs are calculated in LUM and RMS conditions when the neural patterns of responses to NF scenes were decoded by that of LSF or HSF scenes in all 4 categories, only in natural scenes, and only in indoor scenes. The dashed lines indicate chance level. Error bars indicate standard errors; *p < 0.05, **p < 0.01, ***p < 0.001.

When the decoding was performed on the indoor scene categories, one-tailed one-sample *t*-test showed that the decoding accuracies were significantly above chance in all 3 ROIs when the classifiers were trained by the responses to HSF scenes (PPA: *t*(26) = 3.166, *p* = 0.002; RSC: *t*(24) = 2.048, *p* = 0.026; OPA: *t*(26) = 3.850, *p* < 0.001). None of the accuracies was significantly above chance when the classifiers were trained by LSF scenes. Two-tailed paired *t*-tests showed significantly higher decoding accuracies when the NF scenes were decoded by the HSF than by LSF scenes in PPA (*t*(26) = 2.400, *p* = 0.024) and OPA (*t*(26) = 2.759, *p* = 0.010). In the RMS condition, one-tailed one-sample *t*-test showed that the NF scenes could be decoded only by HSF scenes in OPA (*t*(26) = 2.188, *p* = 0.019). Two-tailed paired *t*-tests showed the accuracies of NF scenes decoded by HSF scenes were also significantly higher than that of NF scenes decoded by LSF scenes in OPA (*t*(26) = 5.250, *p* < 0.001). Moreover, the accuracy of training on LSF scenes is significantly lower than the chance level. The decoding accuracies in other conditions were not significant. Two-way ANOVA on the classification accuracies showed significant main effect in SF in all 3 ROIs (PPA: *F*(1, 26) = 10.092, *p* = 0.004; RSC: *F*(1, 24) = 6.384, *p* = 0.019; OPA: *F*(1, 26) = 25.074, *p* < 0.001). No significant interaction was revealed in any of the 3 ROIs. The average classification accuracies in the Cross-SF decoding analysis is showed in Figure 5.

### Behavioral experiment results

In order to confirm that the filtering process did not disrupt the subjects’ abilities to categorize scenes, a separate behavioral experiment was conducted. The classification accuracies were high (above 90%) in all SF conditions in the 2 contrast conditions. In LUM condition, the accuracies were NF: 97.61 ± 3.23%, LSF: 96.48 ± 3.05%, HSF: 95.80 ± 3.05%, and the RT were NF: 580 ± 197 ms, LSF: 591 ± 199 ms, HSF: 595 ± 209 ms. In RMS condition, the accuracies were NF: 95.11 ± 4.19%, LSF: 95 ± 4.15%, HSF: 92.50 ± 3.93%, and the RT were: NF: 613 ± 185 ms, LSF: 606 ± 204 ms, HSF: 600 ± 184 ms. No significant difference in accuracies and RT was found except in RMS condition, the accuracies in NF and LSF were significantly higher than HSF (NF vs. HSF: *t*(21) = 3.279, *p* = 0.004, LSF vs. HSF: *t*(21) = 4.387, *p* < 0.001). The results showed no disruption of scene categorization abilities of subjects by spatial frequency filtering.

## Discussion

In this study, we mainly focus on the representation of the high-level category-specific information in the scene-selective regions when the low-level scene properties was constrained to a certain SF band, as well as the SF preferences in scene perception by performing Cross-SF decoding experiment when the neural patterns of scenes in a specific SF was used to decode the patterns of response to the NF scenes. In addition, we also test the results of the above experimental procedure when the scope of decoding was constrained to indoor or natural scenes and when the luminance contrast of filtered scenes was either unmodified or equalized. Results have shown distinct patterns of SF preferences before and after the contrast equalization, as well as in the different scopes of scenes: the natural and indoor scene categories. The results cast doubts on simply using contrast equalization in spatial frequency analysis and propose that the spatial frequency preferences may differ in different ranges of scenes.

### Distinct SF preferences in overall scene category representations in 2 contrast conditions

First, we performed univariate analysis to investigate the neural activations in different SF condition in LUM and RMS conditions. The results showed different outcomes in the two contrast conditions, indicating higher activations in HSF compared to LSF under RMS conditions. This discrepancy in results might be caused by the differences in presentation time of stimuli and the tasks required for the participants. Then single-SF and cross-SF decoding analyses were performed on all 4 categories to coarsely investigate the SF preferences with and without manipulation of luminance contrast. When the decoding procedure was performed in a single SF, the classification accuracies were supposed to mainly reflect the abilities to distinguish scene categories in a single SF band, such as LSF or HSF. The scenes could be decoded in any SF band in the two contrast conditions. In the decoding on all scene categories, the accuracies did not show significant difference between the 2 SF band, in both contrast conditions. These results suggest that the category information could at least be preserved and processed by the scene-selective regions in a single SF band no matter the contrast is modified or not.

On the other hand, the Cross-SF decoding results mainly reflected the similarities between the patterns of response to LSF or HSF and NF scenes, which can be regarded as the extent to which LSF or HSF information is used in scene understanding in scene-selective regions. In the LUM condition, the NF scenes could be decoded successfully using LSF scenes, whereas the HSF scenes failed to be decoded, particularly under natural scene conditions where the decoding accuracy of HSF scenes was significantly below chance level, despite the presence of single-SF decoding experiment showed that scenes could be successfully decoded in HSF. However, the opposite results were observed in the RMS condition, where the NF scenes could be decoded by the HSF scenes. The NF scenes could only be decoded by LSF scenes in PPA. Based on the findings from facial emotion recognition studies, it is evident that the differential processing of low spatial frequency (LSF) and high spatial frequency (HSF) plays a crucial role in scene category preference. Previous research has shown that luminance contrast alone also accounts for the coarse-to-fine processing of humans, but was weaker compared to the condition when the SF and contrast were both different (Kauffmann et al., 2015a,b). It is stated in a previous study that color contrast in coarse blobs (LSF information) can facilitate human scene segmentation (Oliva and Schyns, 2000). The color contrast may be predominantly revealed in the luminance contrast in our study when the images were conversed to grayscale. We infer that LSF illustrated in previous studies in the perception of coarse scene layout and scene gist (Oliva and Schyns, 2000; Oliva and Torralba, 2006; Schyns and Oliva, 2010) may bind with the high contrast associated in serving these functions, causing the higher accuracies of decoding by LSF scenes in the LUM condition.

After the contrast was increased in the HSF condition, the edges in scenes become more salient, and subjects could more easily interpret the geometric structure of the scenes from the long contours(Walther et al., 2011; Nasr et al., 2014). Therefore, similar to previous studies that used contrast equalization on spatial frequency processing (Peyrin et al., 2004; Berman et al., 2017), the HSF images in RMS condition in our study resemble more to the hand-writing scenes, which showed successful decoding in a previous study (Walther et al., 2011). In addition, the reduction of contrast in the LSF may have disrupted human abilities in perceiving scene spatial structures, causing lower similarity between the neural patterns between LSF and NF. Our results in the RMS condition seem to be consistent with recent single-SF decoding studies showing significant higher accuracies in HSF condition (Berman et al., 2017; Perfetto et al., 2020), because LSF naturally contain more contrast than HSF which leads to not normalizing contrast leaves HSF stimuli at a visible disadvantage. When decoding natural scenes, HSF information acts as a negative interference, and this interference is mitigated under RMS conditions. However, as previous studies have pointed out that contrast values across spatial frequencies follow statistical regularities (Torralba and Oliva, 2003; Guyader et al., 2004), the result after equalizing the luminance contrast in the analysis might not reflect the real scene perception process in the human visual system. Two-way ANOVA on the accuracies in the Cross-SF decoding experiment in the two contrast conditions additionally confirmed the interaction between spatial frequency and contrast. As a result, the contrast might be an indispensable component in spatial frequency processing, and equalizing the luminance contrast should be used with caution in studying human spatial frequency processing.

### SF preferences in decoding of indoor or natural scene categories

Another aspect revealed in the analysis was the different SF preferences in distinguishing indoor and natural scene categories. In the single-SF decoding performed on the natural scene categories, NF and LSF scenes could be successfully decoded, and the accuracies of decoding LSF scenes were significantly higher than decoding HSF scenes in both contrast conditions. Paired *t*-tests regardless of contrast condition also showed significant higher decoding accuracies in LSF compared to HSF. This result indicate that the scene-selective regions are more sensitive to natural scene categories in LSF than HSF. Cross-SF decoding in the condition also showed the successful decoding of NF scenes by LSF scenes in the LUM condition, which substantiated the LSF is mainly used in the representation of natural scene categories in the scene-selective regions. The LSF was suggested to activate peripheral activations in the brain regions (Sasaki et al., 2001; Henriksson et al., 2007), and provide information of global structure and coarse spatial layout of scenes (Schyns and Oliva, 2010; Kauffmann et al., 2015a,b). Natural scene categories, such as beach, mountain and field, usually contain large patches of blobs in similar luminance level, so people tend to recognize their categories from the peripheral vision of global structure rather than foveal vision of details. Consequently, people may preferentially use the LSF information in natural scene perception. As stated in the above section, the high contrast may collaborate with LSF in serving the above functions. However, in the Cross-SF decoding on natural scenes in the RMS condition, the NF scenes could be decoded by HSF scenes in PPA and OPA, and could only be decoded by LSF scenes in PPA. This might because the long contours more saliently reflected the global scene structure after the contrast was increased (Walther et al., 2011), making HSF dominate after the contrast was reduced in LSF which disrupted the global scene perception.

In contrast, when the decoding was constrained to the indoor scenes, the NF scenes could be successfully decoded by the HSF scenes in all ROIs in the LUM condition, and showed significant higher accuracies than decoded by LSF scenes. However, in the RMS condition, the NF scenes could only be decoded by the HSF scenes in PPA, although the single-SF decoding were successful in all ROIs. The results show that when concerning indoor scenes, HSF served a more important role in scene categorization. Unlike the differences between natural scenes in global structure, the indoor scenes have less distinctions in global structure, but more differences in details such as the inner objects. They have been indicated to involve more processing of local 3D space than outdoor scenes (Epstein, 2005; Henderson et al., 2007). Although the long contours in HSF can also represent global structure, the majority of HSF information usually capture the detail information and local space, especially the object shape. Interestingly, the similarities between HSF and NF scenes decreased in the RMS condition. This might be due to the reason that manually adjusting the contrast disturb the SF processing, and cause irrelevant analysis of HSF information which might not be actually contained in the NF scenes.

Therefore, we infer that the differences in SF preferences in natural and indoor scenes may be caused by the different strategies of human beings in categorizing different kinds of scenes. Natural scenes may more be perceived based on the global spatial layouts, and the indoor scenes on the local space details and objects. A previous study has shown the preferences in SF may vary in different kinds of tasks which require local or global processing (Flevaris et al., 2010; Flevaris and Robertson, 2016; Lu et al., 2018). The primary information represented by a specific SF and recognition strategy for scene categories determines which SF is preferentially utilized in perceiving those scenes. The diverse results of neural activations in scene-selective regions revealed in previous univariate studies (Peyrin et al., 2004; Rajimehr et al., 2011; Kreiman and Serre, 2020) might be due to the strategies used in different task demands and presentation time. As a result, the SF processing in human scene perception might be studied in more detail because humans interact with scenes in different ways and perform different tasks in certain scenes.

However, our research still has some limitations. The limited scope of indoor and outdoor scene categories in this study restricts our ability to generalize inferred characteristics. Moreover, selecting a single cutoff frequency for both high and low frequencies limits the spatial frequency resolution, hindering precise evaluation of specific contributions. Behavioral experiments have already explored the SF sampling strategies of observers differ with varying stimuli and task characteristics at higher temporal and spatial frequency resolutions (Wiesmann et al., 2021). Therefore, in further research on the brain processing mechanisms of scene processing, experimental conditions such as increasing spatial frequency resolution, increasing the categories of scene images, and controlling the spatial layout of scene images can be used to further investigate which spatial frequency mainly affects the perception of spatial layout and the specific role of brightness contrast in it.

## Conclusion

In this study, we researched on the SF preferences in human scene perception by investigating the patterns of response when subjects viewed scenes in different SF band. Specifically, we investigated the extent to which each SF information was used in perception of unfiltered scenes. We also examined the effect of contrast equalization on SF processing and the differences in SF preferences between natural and indoor scenes. It is observed that luminance contrast has a significant effect on SF processing of scenes and high luminance contrast may collaborate with LSF information in perceiving the global spatial layout. Furthermore, our observations indicate that natural scene perception primarily relies on LSF information, whereas indoor scenes predominantly rely on HSF information. The distinct SF preferences may be due to the different strategies of global and local perception in different scope of scenes. However, it should be noted that although the Cross-SF decoding was not successful for certain SF in certain conditions, it does not necessarily mean that the corresponding SF in not used in the scene-selective regions. It is possible that the SF that was not successful in Cross-SF decoding may still serve a supplementary role in scene representation, but not provide much high-level information. The results in our study casts doubts on the use of contrast equalization in research on SF processing and suggest the SF processing in visual scene perception should be investigated in a more detailed range.

## Data Availability

The raw data supporting the conclusions of this article will be made available by the authors, without undue reservation.

## Footnotes

^1^ http://www.fil.ion.ucl.ac.uk/spm/software/spm8/
